# *Cytauxzoon felis* Infection in Domestic Cats, Yunnan Province, China, 2016

**DOI:** 10.3201/eid2502.181182

**Published:** 2019-02

**Authors:** Feng-Cai Zou, Zhao Li, Jian-Fa Yang, Jiang-Yan Chang, Guo-Hua Liu, Yan Lv, Xing-Quan Zhu

**Affiliations:** Laboratory of Veterinary Public Health of Higher Education of Yunnan Province, College of Veterinary Medicine, Yunnan Agricultural University, Kunming, China (F.-C. Zou, Z. Li, J.-F. Yang, J.-Y. Chang, Y. Lv);; State Key Laboratory of Veterinary Etiological Biology, Lanzhou Veterinary Research Institute, Chinese Academy of Agricultural Sciences, Lanzhou, China (Z. Li, X.-Q. Zhu);; College of Veterinary Medicine, Hunan Agricultural University, Changsha, China (G.-H. Liu)

**Keywords:** *Cytauxzoon felis*, cytauxzoonosis, cats, China, parasites, vector-borne infections, ITS-2, Yunnan Province, seroprevalence, tickborne disease, domestic cats

## Abstract

We performed a molecular survey for *Cytauxzoon felis* infection in 311 domestic cats in Yunnan Province, China, in 2016 and found a prevalence of 21.5%. *C. felis* infection in domestic and wild cats in other provinces should be investigated to determine parasite prevalence and genetic diversity among cats throughout China.

Cytauxzoonosis is a tickborne hemoprotozoal disease of both domestic cats and wild felids caused mainly by *Cytauxzoon felis* protozoa (*1**,**2*). In the late 1900s, *C. felis* protozoa were reported exclusively in North America, particularly in the mid-Atlantic states of the United States (*3*), but in the early 2000s, this pathogen was reported in some countries of South America, and in Europe, several other *Cytauxzoon* species were identified (*4*). Cytauxzoonosis of domestic cats has long been considered contagious and deadly (*2*). However, as research progressed, the virulence of different *C. felis* isolates was found to be inconsistent; some cats were able to survive the infection and potentially serve as natural reservoirs (*5*).

The number of pet cats around the world is increasing, but the information about the prevalence of *C. felis* infection in domestic cats is limited worldwide. Because of the seriousness of feline cytauxzoonosis and its geographic expansion to more and more regions, informing veterinarians, pet owners, and the general public about this disease has become imperative. The objective of this study was to examine whether *C. felis* infection is present in domestic cats in China.

## The Study

During November–December 2016, we collected whole blood from the femoral vein of 311 domestic cats (74 stray cats and 237 pet cats) in Yunnan Province in southwestern China using EDTA tubes. We stored these EDTA whole blood samples at −20°C and then performed genomic DNA extraction with the TIANamp Genomic DNA Kit (TianGen, http://www.tiangen.com) following the manufacturer’s protocol. To detect *C. felis* infection, we performed a PCR targeting the second internal transcribed spacer (ITS-2) of ribosomal DNA (*6*). We sequenced amplicons in both directions and compared these sequences with those of other relevant *C. felis* isolates available in GenBank. We analyzed differences in *C. felis* prevalence in domestic cats according to lifestyle, region, sex, and age using the χ^2^ test in SPSS 22.0 standard version for Windows (IBM Corporation, https://www.ibm.com). We considered differences statistically significant when the p value obtained was <0.05.

In total, 67 (21.5%) of 311 examined domestic cats were positive for the *C. felis* protozoan. We sequenced these *C. felis*–positive PCR products and obtained 67 ITS-2 sequences; 4 representative sequences were deposited in GenBank (accession nos. MF966369–72). The 67 *C. felis* ITS-2 sequences shared 98.4%–100% similarity. These sequences had 95.6%–100% similarity with corresponding *C. felis* ITS-2 sequences available in GenBank.

The prevalence of *C. felis* protozoa in domestic cats in Yunnan Province was 21.5% (Table), lower than the prevalence in domestic cats in the United States (30.3%, 27/89) (*7*) but higher than that in Brazil (0.66%, 1/151) (*8*). The *C. felis* prevalence in stray cats (51.4%, 38/74) was significantly higher (p<0.001) than that in pet cats (12.2%, 29/237) (Table), probably because stray cats often live outdoors with poor sanitation, thus having high probability of contact with ticks. However, no significant difference in *C. felis* prevalence was found among domestic cats of different sexes or age groups.

**Table:** Prevalence of *Cytauxzoon felis* protozoan in whole blood samples from domestic cats determined by PCR, Yunnan Province, China, 2016

Variable	No. positive/no. tested	Prevalence, % (95% CI)	p value	Odds ratio (95% CI)
Lifestyle				
Stray	38/74	51.4 (40.0–62.7)	<0.001	7.6 (4.2–13.7)
Pet	29/237	12.2 (8.1–16.4)		Referent
Region				
Banna Prefecture	19/38	50.0 (34.1–65.9)	<0.001	10.8 (4.8–24.5)
Honghe Prefecture	5/15	33.3 (9.5–57.2)		5.4 (1.6–17.8)
Lincang City	14/21	66.7 (46.5–86.8)		21.6 (7.6–61.3)
Kunming City	16/189	8.5 (4.5–12.4)		Referent
Yuxi City	13/48	27.1 (14.5–39.7)		4.0 (1.8–9.1)
Sex				
F	38/172	22.1 (15.9–28.3)	0.89	1.1 (0.6–1.9)
M	29/139	20.9 (14.1–27.6)		Referent
Age, y				
<5	21/83	25.3 (15.9–34.7)	0.18	1.8 (0.9–3.4)
5–10	22/133	16.5 (10.2–22.9)		Referent
>10	24/95	25.3 (16.5–34.0)		1.7 (0.9–3.3)
Total	67/311	21.5 (17.0–26.1)		

Distinct *C. felis* genotypes of different virulences in domestic cats have been identified, and genetic diversity among *C. felis* populations has been studied by comparisons of 18S rRNA, ITS-1, and ITS-2 sequences (*1*). ITS-1 and ITS-2 rDNA are better genetic markers for assessing *C. felis* genotypic variability (*9*) because these sequences evolve faster than the 18S rRNA gene. A combination of ITS-1 and ITS-2 sequences has been used to identify the *C. felis* genotypes present in various domestic cats and wild felids (*1*).

*C. felis* protozoa are transmitted to domestic cats by ticks, such as *Amblyomma americanum* and *Dermacentor variabilis* (*1*). Raising pet cats indoors and preventing and treating ectoparasites of outdoor stray cats would help reduce risk for infection in *C. felis* protozoa–endemic areas. Some effective antitick insecticides can be used for preventing transmission of this parasite (*10*).

## Conclusions

Our study revealed a high (21.5%) *C. felis* prevalence in domestic cats in Yunnan Province, China. Further studies are warranted to assess the prevalence of the *C. felis* protozoan in wild felids and domestic cats in other regions of China to estimate its geographic distribution and genetic diversity and to investigate its potential tick vectors.
